# Epigenetics: Recent Advances and Its Role in the Treatment of Alzheimer's Disease

**DOI:** 10.3389/fneur.2020.538301

**Published:** 2020-10-15

**Authors:** Xuewen Xiao, Xixi Liu, Bin Jiao

**Affiliations:** ^1^Department of Neurology, Xiangya Hospital, Central South University, Changsha, China; ^2^National Clinical Research Center for Geriatric Disorders, Central South University, Changsha, China; ^3^Key Laboratory of Hunan Province in Neurodegenerative Disorders, Central South University, Changsha, China

**Keywords:** Alzheiemer's disease, epigenetic, DNA methylation, DNA hydroxymethylation, histone modifications, non-coding RNA (ncRNA)

## Abstract

**Objective:** This review summarizes recent findings on the epigenetics of Alzheimer's disease (AD) and provides therapeutic strategies for AD.

**Methods:** We searched the following keywords: “genetics,” “epigenetics,” “Alzheimer's disease,” “DNA methylation,” “DNA hydroxymethylation,” “histone modifications,” “non-coding RNAs,” and “therapeutic strategies” in PubMed.

**Results:** In this review, we summarizes recent studies of epigenetics in AD, including DNA methylation/hydroxymethylation, histone modifications, and non-coding RNAs. There are no consistent results of global DNA methylation/hydroxymethylation in AD. Epigenetic genome-wide association studies show that many differentially methylated sites exist in AD. Several studies investigate the role of histone modifications in AD; for example, histone acetylation decreases, whereas H3 phosphorylation increases significantly in AD. In addition, non-coding RNAs, such as *microRNA-16* and BACE1-antisense transcript (*BACE1-AS*), are associated with the pathology of AD. These epigenetic changes provide us with novel insights into the pathogenesis of AD and may be potential therapeutic strategies for AD.

**Conclusion:** Epigenetics is associated with the pathogenesis of AD, including DNA methylation/hydroxymethylation, histone modifications, and non-coding RNAs, which provide potential therapeutic strategies for AD.

## Introduction

Alzheimer's disease (AD) is a devastating neurological disease characterized by progressive cognitive impairments. As the most common form of dementia in the world, AD accounts for an estimated 60–80% of dementia cases worldwide (1). It is estimated that 50 million people are living with dementia worldwide currently, and the figure will increase to 152 million by 2050 (2). The common hypotheses of AD include amyloid cascade hypothesis, tau propagation, neuroimmune activation, mitochondrial cascade hypotheses, and infectious hypothesis (3).

AD can be classified into familial and sporadic AD based on family history. Three causative genes, including presenilin 1 (*PSEN1*), presenilin 2 (*PSEN2*), and amyloid precursor protein (*APP*) are involved in the pathogenesis of familial AD in an autosomal-dominant trait (4, 5). *PSEN1* is the most common genetic cause in familial AD, and the second involved gene is *PSEN2. PSEN1* and *PSEN2* encode the presenilins, which are catalytic subunits of BACE1. *APP* is the third involved gene that encodes the APP from which amyloid-β peptide is cleaved. These genetic mutations trigger the cascade of amyloid-β deposition, resulting in cognitive impairments in patients with AD (6, 7).

The genetics of sporadic AD are much more complex than that of familial AD. With the developments of genetic sequencing technology, particularly the Genome-Wide Association Study (GWAS), scientists have identified a number of loci containing susceptibility alleles in sporadic AD. One of the most important loci is the apolipoprotein E gene (*APOE*), and the three major isoforms are *APOE* ε2, ε3, and ε4 based on two-point mutations (rs429358 and rs7412). The *APOE* ε4 haplotype increases the likelihood of the onset of AD (8). Since 2009, tens of GWAS studies on AD have identified more than 20 novel loci including *ABCA7, BIN1, CD33, CLU, CR1, CD2AP, EPHA1, MS4A6A-MS4A4E, PICALM*, and *SORL1*, among others (9, 10).

Currently, much attention has been drawn to the nongenetic factors. Nongenetic factors are of paramount significance in the etiology of AD. Increasing evidence identifies multiple nongenetic factors for AD, most of which are related to lifestyle. A systematic review and meta-analysis based on the current evidence proposes 21 nongenetic factors for the prevention of AD, such as low level of education, hypertension, hyperhomocysteinemia, diabetes, obesity in late life, depression, and stress (11). The underlying mechanisms of how these nongenetic factors affect AD are not fully understood. The common hypotheses include oxidative stress, inflammation, brain reserve theory, the hypoperfusion hypothesis, and hypomethylation theory (12). In the 5xFAD mouse model, environmental enrichment, a combination of cognitive and sensory stimulation and social interaction, improves cognitive impairment via altering epigenetic markers (13).

**Figure 1:**
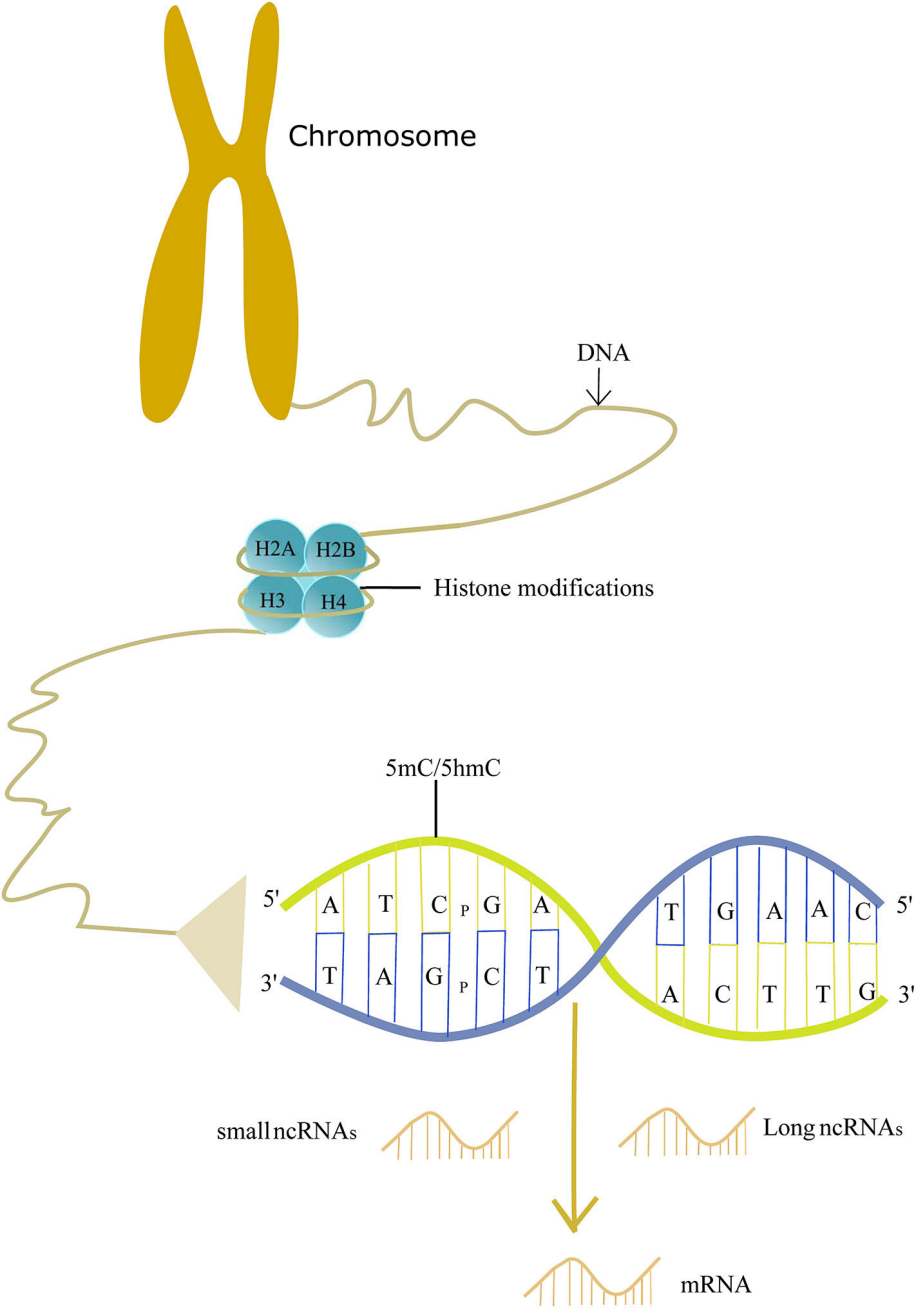
Schematic picture of epigenetics in the pathogenesis of AD.

Epigenetics may explain the roles of non-genetic factors involved in AD and help us to better understand the etiology of AD. Epigenetics was so named first by Conrad Waddington in the 1940's, and it is now defined as the study of molecules and mechanisms that perpetuate alternative gene activity states without changing the DNA sequence (14). Specifically, epigenetics includes DNA methylation/hydroxymethylation, histone modifications, and non-coding RNA regulation. These modifications play a crucial role in the gene readout, such as gene silencing, transcription, and post-transcriptional RNA processing. Therefore, epigenetics makes a significant impact on the disease (15, 16). Evidence shows that epigenetics is involved in the pathogenesis of AD (Figure 1). Here, we review recent findings on the epigenetics of AD and its potential therapeutic strategies.

## DNA Methylation/Hydroxymethylation in AD

DNA methylation refers to methylation of the nucleobases in DNA by a set of proteins called DNA methyltransferases (DNMTs). Most of the methylated nucleobases are cytosines in CpGs and CpHs (H = A, T, C), forming 5-methylcytosine (5 mC). Five mC is mostly enriched in CpG dinucleotides (17). Although a lot of attention on DNA methylation focuses on CpGs, CpHs methylation is found to be associated with gene expression in neurons (18). In addition to 5 mC, methylation of another nucleobase is identified. In mouse prefrontal cortical neurons, deoxyadenosine methylation on N6 (m6dA) is correlated with gene expression and the formation of memory (19). DNMTs include DNA methylTransferase 1, DNA methylTransferase 3A, and DNA methylTransferase 3B (20). DNA hydroxymethylation is the hydroxymethylation of the cytosine to develop 5-hydroxymethylcytosine (5 hmC), and 5 hmC is converted from 5 mC by ten-eleven translocase (TET) isoforms (21). CpG and CpH are abbreviations for the cytosine and other nucleobases divided by a phosphate. The methyl/hydroxymethyl groups are located in the major groove of the DNA helix where many DNA-binding proteins make contact. Therefore, DNA methylation can lead to gene silencing, X-chromosome inactivation, and genomic imprinting. The exact functions of DNA hydroxymethylation remain unclear, and it may promote or repress gene expression by binding to regulatory regions of a gene (22, 23).

To date, a variety of studies have analyzed the role of DNA methylation in AD. One of the most studied genes of DNA methylation is the *APP* gene. CpG dinucleotides are enriched in the 5′ region of the *APP* gene, and alteration of its methylation levels can affect APP expression. Some studies identify hypomethylation of the *APP* gene in patients with AD compared with normal controls by analyzing postmortem brains or peripheral blood leucocytes *in vitro* (24–26), whereas, two other studies show no differences between AD and normal controls (27, 28). The reasons for the different results are not well-understood. Possible explanations include different test methods, different tissues examined, and relatively small sample sizes (29). Specifically, these studies examine the *APP* methylation levels in postmortem brain tissues or blood from a few AD patients and normal controls. Large-scale studies on methylation of the *APP* gene may address such different results in the future. Meanwhile, by analyzing genomic DNA, there are markedly different methylation levels of the *APP* gene in different human tissues (30). Therefore, the different tissues examined may also account for the different findings in the *APP* methylation levels. Furthermore, methylcytosines are reduced in the *APP* gene with age, which may be associated with Aβ deposition in AD (31). In addition to age, gender affects the methylation levels of the *APP* gene. Hypermethylation of the *APP* gene is identified in the female mouse cerebral cortex compared to male mice (32).

*PSEN1* and *PSEN2* genetic methylation patterns do not differ significantly between AD samples and normal controls (27, 28). After adjusting for gender and *APOE*, there are no significant methylation levels of *PSEN1* and *PSEN2* between AD patients and normal controls (33). No difference in *PSEN1* methylation levels is observed in the AD mouse model, suggesting that Aβ production is not associated with *PSEN1* methylation (34). Nevertheless, reduced methylation of the *PSEN1* gene is identified in the human cerebral cortex (35, 36). In blood DNA, *PSEN1* methylation is significantly downregulated in AD patients compared to normal controls, which is associated with higher *PSEN1* expression during the progression of AD (37). The inconsistent findings may be associated with significant interindividual methylation variance of the *PSEN1* gene (38). For *APOE*, two studies show that there is no significant difference in DNA methylation levels in postmortem brain tissues between AD patients and normal controls (39, 40). However, Foraker et al. observe a significant decrease in DNA methylation levels of *APOE* by evaluating methylation profiles of AD postmortem brains *in vitro* (41). The reason for this inconsistency may result from the detection platform. The previous two studies mainly focus on *APOE* promoter regions lacking CpG islands based on bead-chip. In the subsequent study, CpG methylation of *APOE* is obtained by pyrosequencing, which is more reliable in reflecting *APOE* methylation levels (41). Another study demonstrates that non-neuronal cells mainly contribute to the low levels of *APOE* DNA methylation in AD patients (42).

**Figure 2:**
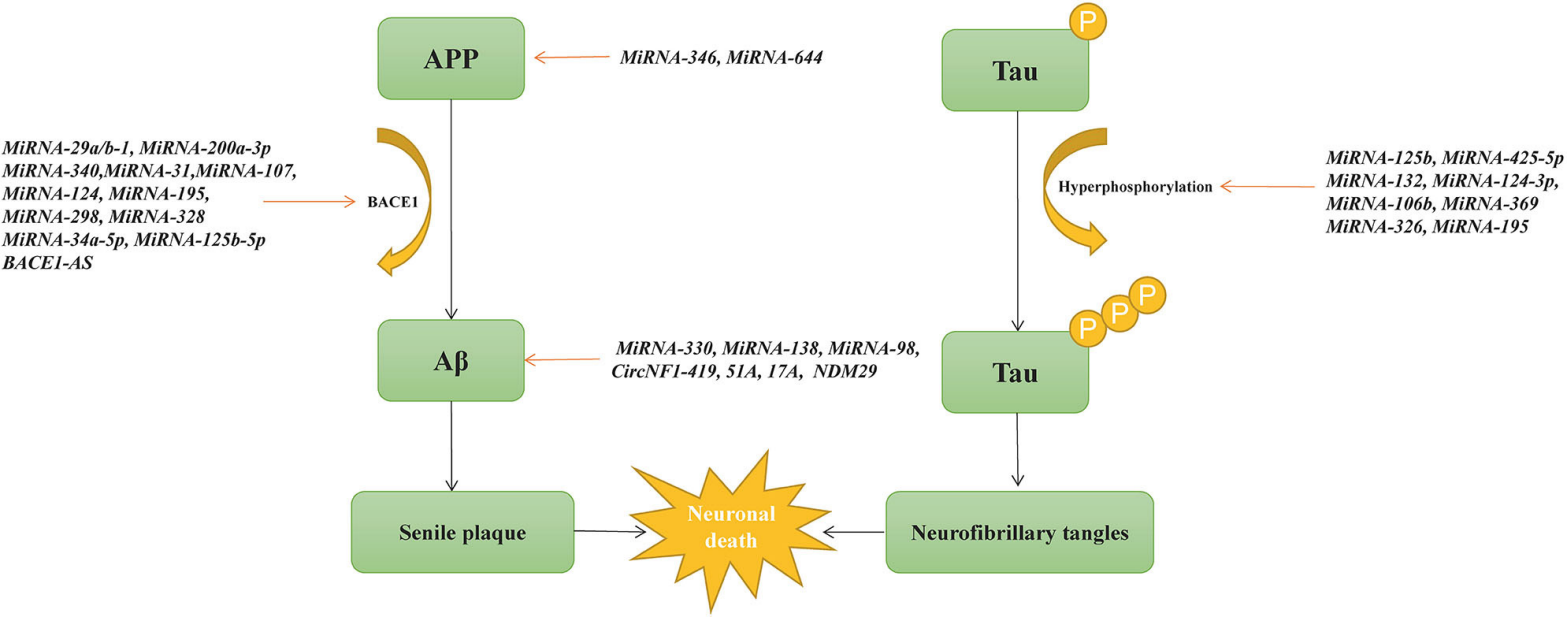
Specific gene methylation in AD: candidate gene approach.

Further, a lot of studies examine methylation sites of candidate susceptibility genes for AD (Figure 2). An et al. show that the 2′,5′-oligoadenylate (2-5A) synthetase gene is hypomethylated in AD cells *in vitro* (43). Sanchez-Mut et al. show that *TBXA2R, SORBS3*, and *SPTBN4* are hypermethylated in 12 distinct AD mouse brain regions, suggesting that the axon initial segment and cAMP response element-binding protein (CREB) activation pathway is involved in AD pathogenesis (44). Lei Yu et al. demonstrate that DNA methylation levels in *SORL1, ABCA7, HLA-DRB5, SLC24A4*, and *BIN1* are associated with greater odds for pathological AD by examining 28 reported AD loci discovered from recent GWAS reports on AD, which indicate that the change of DNA methylation of AD risk genes contribute to the etiology of AD (45). The hypermethylation of *BDNF* is repeatedly observed in AD patients' blood samples, which might be a diagnostic marker of AD (46, 47). Nevertheless, Carboni et al. find that *BDNF, SIRT1*, and *PSEN1* exhibit no different methylation patterns in AD compared with controls using peripheral blood samples *in vitro*. The inconsistency among the studies may result from methodological differences. Bisulphite pyrosequencing technology and methylation-specific primer real-time PCR cover different CpGs when evaluating CpG sites in *BDNF* (48).

Fabio Coppedè et al. show that there are no methylation differences in DNA repair genes between patients with AD and non-affected individuals, including *OGG1, PARP1, MRE11A, BRCA1, MLH1*, and *MGMT* (49). Ma et al. find that *UQCRC1* is highly methylated in patients with AD by studying peripheral blood samples of AD, suggesting that inflammation and oxidative stress may contribute to AD (50). The DNA methylation level of *PLD3* is increased and correlated with hippocampal Aβ in AD hippocampus compared to controls (51). A lower DNA methylation level at *TREM2* is observed in AD patients and associated with TREM2 mRNA expression, which may be a biomarker for AD (52). *CRTC1* is hypomethylated in the AD hippocampus and associated with p-tau deposition (53). Hypomethylation of CpGs in *BIN1* is identified in participants with AD and confers risk to AD by studying peripheral blood, indicating it may be a biomarker for AD (54). The methylation level of *BIN1* is associated with neuritic plaque pathology in the peripheral blood (55). Furthermore, the methylation levels of *OPRM1* and *OPRL1* are significantly increased in AD compared to controls, suggesting that opioid receptor genes may be potential biomarkers for diagnosing AD (56). The DNA methylation level of *PICALM* is decreased and linked to cognitive decline in AD (57). Elevated *ANK1* DNA methylation exists in the entorhinal cortex of AD and is associated with AD pathology (58). Similarly, another study also identifies that hypermethylated *ANK1* is observed in the entorhinal cortex, superior temporal gyrus, and prefrontal cortex (39). Methylation of *ABCA2* is negatively associated with AD risk and may be a therapeutic target of AD (59). Furthermore, other studies identify many genes without methylation changes between AD and normal controls, such as *SORL1, SIRT1, SST, SSTR4, HSPA8, HSPA9, SIRT3*, and *ABCA7* (40, 60–63) (Table 1).

**Table 1:** Specific gene methylation and hydroxymethylation in Alzheimer's disease: candidate gene approach.

**Gene**	**Function**	**Tissue type**	**Main finding**	**References**
*APP*	Amyloid precursor protein	Human postmortem cerebellum, parietal lobe and temporal lobe	Hypomethylation	(24)
*APP*	Amyloid precursor protein	Human postmortem temporal lobe	Hypomethylation	(25)
*APP*	Amyloid precursor protein	Human peripheral blood samples	Hypomethylation	(26)
*APP/PSEN1/PSEN2*	Amyloid precursor protein/component of γ-secretase	Human postmortem frontal cortex and hippocampus	No differences	(27)
*APP/PSEN1/PSEN2*	Amyloid precursor protein/component of γ-secretase	Human postmortem frontal cortex, parietal cortex, temporal cortex, and cerebellum	No differences	(28)
*PSEN1/PSEN2*	Component of γ-secretase	Human peripheral blood	No differences	(33)
*PSEN1*	Component of γ-secretase	Human postmortem cerebral cortex	Hypomethylation	(35)
*PSEN1*	Component of γ-secretase	Human postmortem cerebral cortex	Hypomethylation	(36)
*PSEN1*	Component of γ-secretase	Human peripheral blood	Hypomethylation	(37)
*APOE*	Risk gene for AD	Human postmortem entorhinal cortex, cerebellum, superior temporal gyrus and prefrontal cortex	No differences	(39)
*APOE*	Risk gene for AD	Human postmortem brain tissues (entorhinal and auditory cortices and hippocampus)	No differences	(40)
*APOE*	Risk gene for AD	Human postmortem cerebellum, hippocampus	Hypomethylation	(41)
*2′,5′-oligoadenylate (2-5A) synthetase*	An enzyme induced by interferon (IFN)	Human skin fibroblasts	Hypomethylation	(43)
*SORL1, ABCA7, HLA-DRB5, SLC24A4*, and *BIN1*	Risk gene for AD	Human dorsolateral prefrontal cortex tissue	Their methylation was associated with AD pathologies	(45)
*BDNF*	Nerve growth factor	Human peripheral blood samples	Hypermethylation	(46)
*BDNF*	Nerve growth factor	Human peripheral blood samples	Hypermethylation	(47)
*BDNF, SIRT1*, and *PSEN1*	Nerve growth factor/Sirtuin 1/ Component of γ-secretase	Human peripheral blood samples	No difference	(48)
*OGG1, PARP1, MRE11A, BRCA1, MLH1*, and *MGMT*	DNA repair	Human peripheral blood samples	No differences	(49)
*UQCRC1*	A subunit of the respiratory chain protein	Human peripheral blood samples	Hypermethylation	(50)
*PLD3*	Risk gene for AD	Human hippocampal samples	Hypermethylation	(51)
*TREM2*	Risk gene for AD	Human peripheral blood samples	Hypomethylation	(52)
*CRTC1*	CREB regulated transcription coactivator 1	Human postmortem hippocampus	Hypomethylation	(53)
*BIN1*	Risk gene for AD	Human peripheral blood samples	Hypomethylation	(54)
*OPRM1* and *OPRL1*	Opioid receptor genes	Human peripheral blood samples	Hypermethylation	(56)
*PICALM*	Risk gene for AD	Human peripheral blood samples	Hypomethylation	(57)
*ANK1*	Encoding for ankyrin-1	Human postmortem entorhinal cortex	Hypermethylation	(58)
*ABCA2*	Risk gene for AD	Human peripheral blood samples	Methylation of *ABCA2* was negatively associated with AD risk	(59)
*SORL1* and *SIRT1*	Risk gene for LOAD	Human postmortem brain tissues (entorhinal and auditory cortices and hippocampus) and peripheral blood leukocytes	No difference	(40)
*SST* and *SSTR4*	Somatostatin and its receptor	Human postmortem brain tissue (middle temporal and superior frontal gyrus)	No difference	(60)
*HSPA8* and *HSPA9*	Chaperone	Human postmortem brain tissue (entorhinal and auditory cortices and hippocampus) and peripheral blood samples	No difference	(61)
*PSEN1,BACE1,MTHFR, DNMT1, DNMT3A*, and *DNMT3B*	*PSEN1* and *BACE1*: Aβ production *MTHFR*: one-carbon metabolism *DNMT1, DNMT3A*, and *DNMT3B*:DNA methylation	Human peripheral blood samples	No difference	(62)
*ABCA7*	Risk gene for AD	Human peripheral blood samples	No differences	(63)
*TREM2*	Risk gene for AD	Human postmortem hippocampus	Hyperhydroxymethylation	(64)
*TBXA2R/SORBS3/SPTBN4*	Family of G protein-coupled receptors/involved in synapsis/member of the axon initial segment	C57BL/6J mice and human postmortem frontal cortex	Hypermethylation	(44)
*PSEN1*	Component of γ-secretase	TgCRND8 mice brains and blood	No differences	(34)

*BDNF, brain derived neurotrophic factor; SIRT1, Sirtuin 1; OGG1, 8-Oxoguanine DNA Glycosylase; PARP1, Poly-ADP-ribose polymerase-1; MRE11A, Meiotic Recombination 11 Homolog A; BRCA1, breast cancer 1; MLH1, MutL homolog 1; MGMT, O6-methylguanine-DNA methyltransferase; UQCRC1, Ubiquinol-Cytochrome C Reductase Core Protein 1; PLD3, phospholipase D family member 3; TREM2, Triggering receptor expressed on myeloid cells 2; CRTC1, CREB Regulated Transcription Coactivator 1; OPRM1, Opioid Receptor Mu 1; OPRL1, Opioid Related Nociceptin Receptor 1; PICALM, Phosphatidylinositol Binding Clathrin Assembly Protein; ANK1, Ankyrin 1; ABCA2, ATP Binding Cassette Subfamily A Member 2; SST, Somatostatin; SSTR4, Somatostatin Receptor 4; HSPA8, Heat Shock Protein Family A (Hsp70) Member 8; HSPA9, Heat Shock Protein Family A (Hsp70) Member 9; MTHFR, Methylenetetrahydrofolate Reductase; DNMT1, DNA Methyltransferase 1; DNMT3A, DNA Methyltransferase 3 Alpha; DNMT3B, DNA Methyltransferase 3 Beta*.

Recently, there were several genome-wide methylation analyses of AD. By examining genome-wide enhancer methylation levels in the prefrontal cortex of AD patients, Li et al. identify 1,224 enhancer regions methylated differentially and most of their methylation levels are decreased in AD neurons, which are associated with Aβ, tau, and cognitive impairment (65). Humphries et al. show that 1,106 of 5,147 CpG sites differ between LOAD patients and controls, 87.3% of which are hypomethylated and related to the myelination network in LOAD (66). Moreover, Watson et al. identify 479 differentially methylated regions in patients with AD compared to normal controls by performing a genome-wide screen of DNA methylation in the temporal gyrus of AD, and 475 of these regions are involved in neuron function and development (67). De Jager et al. show that 11 of 415,848 interrogated CpGs are significantly associated with the AD pathological burden by studying AD autopsied brains, including CpGs in *ABCA7* and *BIN1* regions (68). Moreover, an epigenome-wide study identifies that DNA methylation of *OXT* is associated with the risk of AD, indicating it may be a novel promising biomarker or therapeutic target in AD (69). In another epigenome-wide DNA methylation study, among 17,895 differentially methylated CpG sites, there are 11,822 hypermethylated CpGs and 6,073 hypomethylated CpGs in the superior temporal gyrus of AD patients (70). When examining 420,852 DNA methylation sites from four brain regions in late-onset AD and neurotypical controls, 858 sites show differential methylation patterns, indicating that DNA methylation may contribute to AD (71). Altuna et al. profile genome-wide DNA methylation levels in the hippocampus of AD patients in which 118 AD-related differentially methylated positions are found, and these positions are linked to phosphorylated tau burden (72). The first integrated base-resolution genome-wide study finds 39 CpG site-specific and 27 AD region-specific epigenetic changes in AD, providing reliable epigenetic signatures for the diagnosis and treatment of AD (73) (Table 2).

**Table 2:** Specific gene methylation and hydroxymethylation in AD: Genome-wide approach.

**Design and cases**	**Tissue type**	**Methylation sites**	**Main finding**	**References**
101 individuals with no/mild, moderate and severe AD pathology (Braak stage: 1–2 *n* = 38 individuals, 3–4 *n* = 32, and 5–6 *n* = 31 individuals, respectively)	Human postmortem prefrontal cortex	1.2 million CpG and CpH sites in enhancers	1,224 differentially methylated enhancer regions; most of which are hypomethylated at CpH sites in AD neurons	(65)
AD cases (*N* = 10) compared to normal controls (*N* = 10) and disease controls (DLB *N* = 10)	Human postmortem temporal pole	5,147 CpG sites on 465 genes	1,106 of the 5,147 CpG sites differed between LOAD patients and controls, and 87.3% of them was hypomethylated in LOAD	(66)
AD patients (*N* = 34) and non-AD subjects (*N* = 34)	Human postmortem superior temporal gyrus	461,272 autosomal CpGs	479 differentially methylated regions in AD patients, and 475 of these regions are involved in neuron function, metabolism and development	(67)
Investigating AD methylation state with the burden of AD pathology prospectively (N=708)	Human postmortem dorsolateral prefrontal cortex	415,848 interrogated CpGs	11 of 415,848 interrogated CpGs are significantly associated with AD pathological burden, including CpGs in the *ABCA7* and *BIN1* regions	(68)
Comparison of AD patients (45) with age-matched controls (*N* = 35) and converters to AD dementia (N=54) and non-converters (*N* = 42)	Human postmortem middle temporal gyrus and peripheral blood samples	Epigenome-wide patterns of DNA 5 mC and 5 hmC	DNA methylation of *OXT* was associated with AD risk in the elderly	(69)
34 patients with late-onset AD and 34 controls without dementia	Human postmortem superior temporal gyrus	17,895 differentially methylated CpG sites	There were 11,822 hypermethylated CpGs and 6,073 hypomethylated CpGs	(70)
Late-onset AD patients (*N* = 24) and controls (*N* = 49)	Human postmortem hippocampus, entorhinal cortex, dorsolateral prefrontal cortex and cerebellum	420,852 DNA methylation sites	858 sites showed differential methylation patterns	(71)
26 AD patients and 12 control subjects	Human postmortem hippocampal samples	5-methylcytosine	118 AD-related differentially methylated positions were identified	(72)
371 AD patients and 163 control subjects	Normal and AD patient derived iPSCs, neural progenitor cells, and cortical neuronal cells	5-methyl-cytosine (5mC), 5-hydroxymethyl-cytosine (5 hmC), and 5-formyl/carboxy-cytosine (5fC/caC)	39 CpG site-specific and 27 AD region-specific epigenetic changes	(73)
Identifying AD DhMRs associated with AD pathology (*N* = 30)	Human postmortem dorsolateral prefrontal cortex tissue	5-hydroxymethylcytosine (5 hmC) at specific genomic loci	517 DhMRs significantly associated with neuritic plaques while 60 DhMRs associated with neurofibrillary tangles	(74)
Comparison of AD (*N* = 6) with control cases (*N* = 5)	Human postmortem frontal cortex	5-methylcytosine and 5-hydroxymethylcytosine (5 hmC)	There were 325 genes containing differentially hydroxymethylated loci in AD	(75)
Late-onset AD (LOAD) (*n* = 5) and neurologically normal controls (*n* = 5)	Human postmortem frontal cortex tissues	16,165 DhMRs annotated to 8,149 genes	HIF2 α and HIF1α was enriched in the DhMRs	(76)
96 individuals	Human postmortem cortex tissues	5-methylcytosine and 5-hydroxymethylcytosine (5 hmC)	Hypohydroxymethylation in ANK1 was found in entorhinal cortex of AD patients	(77)

*DLB, Dementia with Lewy bodies; ATP, binding cassette subfamily A member 7; BIN1, box-dependent-interacting protein 1; OXT, Oxytocin; DhMRs: differentially hydroxymethylated regions; HIF2 α, hypoxia-inducible factor 2α; HIF1α, hypoxia-inducible factor 1α; ANK1, Ankyrin 1. Hypohydroxymethylation in ANK1 was found in entorhinal cortex of AD patients*.

Global DNA methylation/hydroxymethylation is the percentage of methylcytosine/hydroxymethylcytosine of total cytosine. The percentage of methylation of CCGG sites exhibits no significant difference by investigating DNA methylation in the human brain (78). Moreover, no significant global 5mC and 5hmC changes are found in the entorhinal cortex of AD patients (79). Some studies find that the levels of global 5 mC and 5 hmC is decreased in patients with AD (80–82). However, in the postmortem AD cortex, global levels of 5 mC and 5 hmC are significantly increased in AD samples and correlated with biomarkers of AD (83). Global increased 5 mC levels are identified in the postmortem frontal cortex from AD patients (84). By analyzing DNA isolated from peripheral blood, global DNA methylation levels are upregulated in AD patients and correlated with cognitive impairments (85). The reasons for the difference are far from being understood. One possible reason is that they investigated different brain regions. Nevertheless, some findings are contradictory even for the same test method and brain region. The differences in test method procedures and sample size may account for the different findings. In addition to studies in postmortem brain regions, global DNA methylation/hydroxymethylation was also investigated in the AD mouse model. The 5xFAD mouse model is composed of two *APP* and two *PSEN1* mutations. Global high levels of 5-mC and a reduction of 5-hmC were observed in the 5xFAD mouse model, which are related to cognition and paralleled with Aβ deposition (86). In the 3xTg-AD mouse model, global methylation and hydroxymethylation levels are reduced (87). In the SAMP8 mouse model, global DNA methylation levels are decreased while 5-hmC levels are upregulated (88).

Compared to DNA methylation, relatively few studies analyze the role of DNA hydroxymethylation in AD. As we mentioned previously, 5 hmC is the specific product of DNA hydroxymethylation. Five hmC is highly concentrated in the central nervous system and plays an important role in neurodevelopment and neurological function (89, 90). The triggering receptor expressed on myeloid cells (TREM2) is a receptor in brain microglia and contributes to the pathogenesis of AD (91). When examining DNA hydroxymethylation levels in *TREM2*, Celarain et al. find that the 5 hmC levels were increased in AD patients and involved in TREM2 mRNA expression (64) (Table 1). Shu et al. demonstrate that the 5 hmC levels are decreased in the hippocampus in a mouse model of AD, whereas another study finds that the 5 hmC levels are significantly increased in it in AD patients. Different tissue types may explain the contradictory findings (92, 93). The genome-wide distribution of 5 hmC finds that 517 differentially hydroxymethylated regions (DhMRs) are significantly associated with neuritic plaques, whereas 60 DhMRs are associated with neurofibrillary tangles (74). Moreover, there are 325 genes containing differentially hydroxymethylated loci in AD from genome-wide analyses of 5 hmC in the prefrontal cortex of postmortem AD patients, involving neuronal projection development and neurogenesis (75). By studying genome-wide profiles of 5 mC and 5 hmC profiles in frontal cortex tissues from Chinese AD patients, two significant transcription factor-binding motifs, hypoxia-inducible factor 2α, and hypoxia-inducible factor 1α are enriched in the differentially hydroxymethylated regions (76). As mentioned previously, DNA hypermethylation in *ANK1* is identified in AD (58). Hypohydroxymethylation in *ANK1* is also found in the entorhinal cortex of AD patients in an epigenome-wide association study, further highlighting the significant role of *ANK1* in the development of AD (77) (Table 2).

It is increasingly becoming accepted that mitochondria play a significant role in the pathogenesis of AD (94). Blanch et al. show that mitochondrial 5 mC levels increase, whereas no significant differences in 5 hmC levels are identified in AD (95). However, another study finds that mitochondrial 5 hmC is significantly increased in the superior temporal gyrus of AD subjects (93). The different test methods among these studies may account for the different findings of mitochondrial 5 hmC in brain tissue. In APP/PS1 transgenic mice, the displacement loop mitochondrial methylation level is reduced while *12 S rRNA* gene mitochondrial methylation is increased, indicating that mitochondrial DNA methylation may play a role in the AD development (96).

## Histone Modifications in AD

Histone is a kind of octamer consisting of pairs of H2A, H2B, H3, and H4, which form the nucleosome with DNA. Histone can be modified at the N-terminal tails, and the modifications can affect the three-dimensional structure of the chromatin, leading to the changes in the transcription of genes. The common histone modifications include acetylation, methylation, and ubiquitination. The modifications are controlled by a specific set of enzymes, such as acetyltransferases and deacetylases (97).

To date, there have been several studies of histone modifications in AD. A genome-wide study of H3K27 acetylation in AD observed 4,162 differential acetylomic variation peaks between AD patients and normal controls, which were associated with Aβ and tau pathology (98). Zhang et al. show that histone acetylation is significantly decreased in the temporal lobe of patients with AD compared with that of controls, which is consistent with the previous finding in an APP/PS1 mouse model of AD, highlighting that histone acetylation is involved in AD (99, 100). Narayan et al. demonstrate that acetyl histone H3 and H4 levels are significantly increased in postmortem AD brain tissue compared with normal controls by investigating global acetyl histone levels (101). Another study shows a significant increase of monocytic H4K12 acetylation in transgenic AD mouse models and MCI patients (102). Global histone H3 acetylation levels exhibit a significant increase in the frontal cortex in end-stage AD patients, supporting that histone acetylation plays an important role in AD (103). These studies highlight that histone acetylation levels are increased on a global scale in the AD brain. Anderson et al. observe notable decreases in methylation of H2B and H4 residues, whereas, the ubiquitination of H2B residue increases. These post-translational histone modifications are related to AD pathology and of great significance in the development of AD (104). With regard to the regulators of histone modifications, in APP/PS1 mice, age is associated with the global levels of histone modifications, suggesting that age is one of the main risk factors in the histone modifications of AD (105). An epigenome-wide association study demonstrates that tau protein affects histone acetylation changes and an altered chromatin structure in AD prefrontal cortices (106) (Table 3).

**Table 3:** Histone modifications in AD.

**Histone modifications**	**Tissue type**	**Main findings**	**References**
H3K27 acetylation	Human postmortem entorhinal cortex samples	4,162 differential acetylomic variation peaks between AD and normal controls	(98)
Histone acetylation	Human postmortem temporal lobe	Decreased significantly in AD	(99)
H3 and H4 acetylation	Human postmortem temporal gyrus	Increased significantly in AD	(101)
H3 acetylation	Human postmortem frontal cortex	Increased substantially in AD	(103)
H4 acetylation	Human postmortem frontal cortex	Decreased significantly in AD	(104)
H2B ubiquitination	Human postmortem frontal cortex	Increased significantly in AD	(104)
Histone acetylation	Hippocampus of AD APP/PS1 mouse	Decreased significantly in AD	(100)
H4K12 acetylation	Transgenic AD mouse models and MCI/AD patients monocytes	Increased significantly in AD	(102)

*H3K27, histone H3 at lysine 27; H2B, histone 2B; H3, histone H3; H4, histone H4; H4K12: histone H4 at lysine 12; MCI, mild cognitive impairment*.

## Non-Coding RNAs in AD

Non-coding RNAs (ncRNAs) are defined as RNA molecules that are not translated into a protein. Less than 2% of the human genome encodes proteins, and the rest produces thousands of ncRNAs, including small ncRNAs (smaller than 200 nucleotides in length), such as microRNAs, small interfering RNAs, piwi-interacting RNAs, and a variety of long ncRNAs (longer than 200 nucleotides) (107).

**Figure 3:**
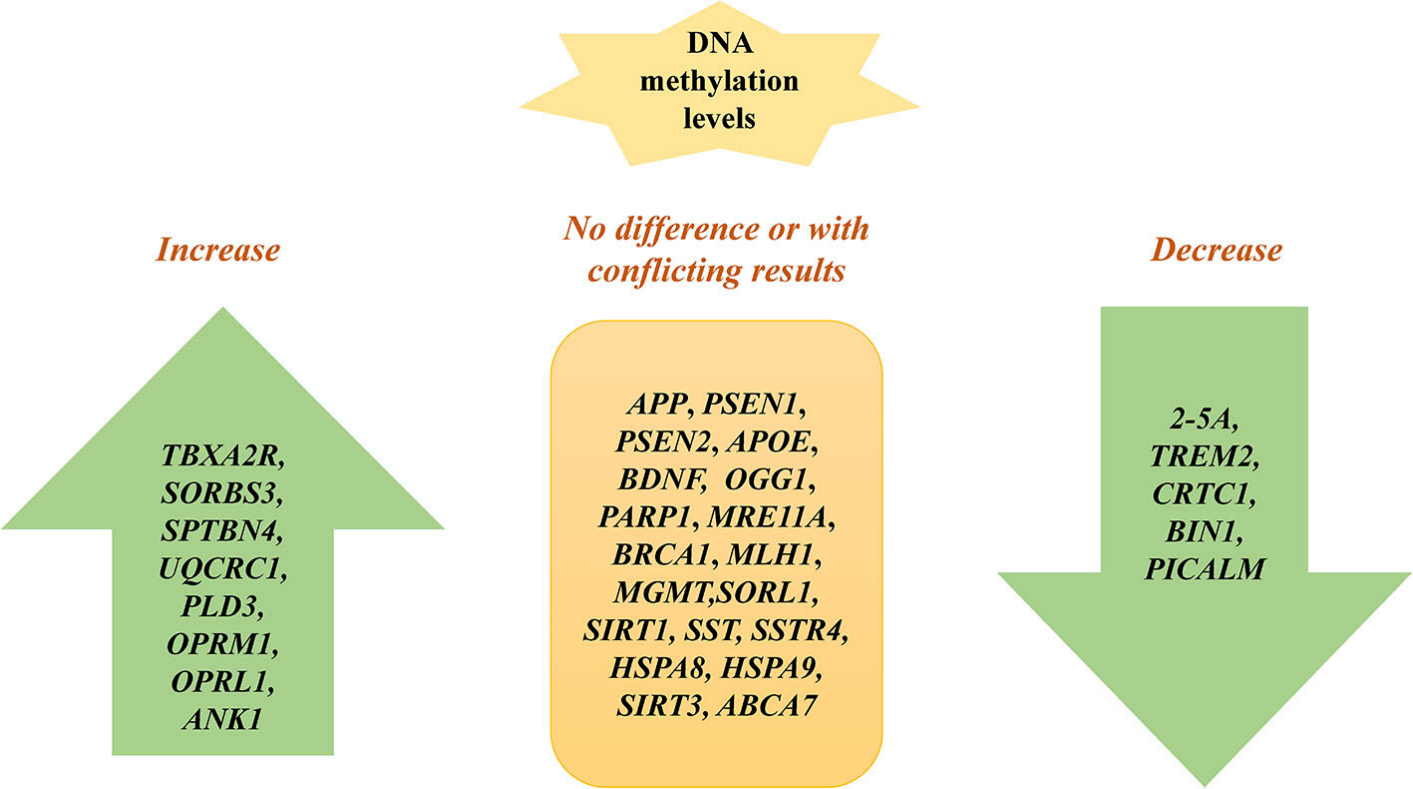
Non-coding RNAs implicated in Aβ and tau protein metabolism.

One of the best characterized small ncRNAs in AD is microRNA. Many microRNAs are surrounded by the CpG island and located within exons, intergenic regions, or introns of genes (108). MicroRNAs can enhance or repress messenger RNA transcription by binding to the 3′-untranslated region or the promoter of the target gene (109). In sporadic AD, microRNAs play an important role in Aβ production and neurofibrillary tangle formation (Figure 3). Two studies demonstrate that *microRNA-16* inhibits the expression of APP both *in vitro* and *in vivo*, which may be a therapeutic target of AD (110, 111). As mentioned previously, BACE1 is one of the APP-cleaving enzymes in the production of Aβ. By investigating changes in 328 *microRNA* expression profiles, Hébert et al. find that *microRNA* cluster *microRNA-29a*/*b-1* suppressed endogenous BACE1 expression, and it is significantly decreased in sporadic AD patients (112). *MicroRNA-200a-3p* also reduces the expression of BACE1 and is confirmed to be decreased in the hippocampus of APP/PS1 and SAMP8 mice as well as in blood plasma from AD patients (113). *MicroRNA-340* is decreased in the hippocampus of an AD mouse model and associated with the overproduction of Aβ by targeting BACE1 (114). *MicroRNA-31* reduces the mRNA levels of BACE1 and improves memory deficits in AD triple-transgenic (3xTg-AD) female mice (115). Moreover, *microRNA-107, microRNA-124, microRNA-195, microRNA-298*, and *microRNA-328* are also associated with the expression of BACE1 (116–119). Another recent study shows that *microRNA-298* represses the expression of BACE1, APP, Aβ40, and Aβ42 in the cell model, suggesting that *microRNA-298* may be a therapeutic target for AD (120). Meanwhile, the addition of *microRNA-34a-5p* and *microRNA-125b-5p* reduces Aβ by targeting BACE1 (121). Abnormally phosphorylated tau protein is another key pathological hallmark of AD. Li et al. find that *microRNA*−*219-5p* is significantly upregulated in brain tissues of AD patients, contributing to tau phosphorylation and AD progression (122). *MicroRNA-125b* promotes the phosphorylation of tau by activating cyclin-dependent kinase 5 (CDK5) and p35/25, and *microRNA-125b* is increased in AD patients (123). Similarly, overexpression of *microRNA-125b* results in tau hyperphosphorylation by targeting phosphatases DUSP6 and PPP1CA (124). In HEK293/tau cells, *microRNA-425-5p* overexpression promotes tau phosphorylation through targeting HSPB8 in AD (125). *MicroRNA-132*, the most significantly downregulated microRNA in AD, is associated with lower levels of tau phosphorylation by regulating EP300, GSK3b, Rbfox1, proteases Calpain2, and caspases 3/7 (126). *MicroRNA-124-3p* inhibits abnormal tau hyperphosphorylation through targeting the Caveolin-1-PI3K/Akt/GSK3β pathway in AD (127). *MicroRNA-106b* reduces tau phosphorylation induced by Aβ42 by involving the expression of Fyn (128). In 3xTg-AD mice, knocking out *microRNA-369* is associated with tau hyperphosphorylation via regulating Fyn and SRPK2 signaling pathways (129). *MicroRNA-326* decreases tau hyperphosphorylation and improves cognitive functions of AD through the JNK signaling pathway (130). Elevating the levels of *microRNA-195* diminishes tau hyperphosphorylation and Aβ burden in ApoE4^+/+^ mice (131).

Furthermore, some altered microRNAs are directly related to APP or Aβ processing in AD. *MicroRNA-346* upregulates APP translation and Aβ production by binding to *APP* 5′UTR, and its levels change in late-stage AD patients (132). *MicroRNA-644* downregulate the formation of Aβ via targeting *APP* 3'UTR (133). Overexpression of *microRNA-330* reduces Aβ production and alleviates oxidative stress through the MAPK signaling pathway (134). In APP/PS1 mice, *microRNA-138* enhances Aβ production and improves cognitive impairment by decreasing the expression of sirtuin 1 protein (135). *MicroRNA-98* improves oxidative stress and downregulates Aβ production by activating the Notch signaling pathway (136). In addition to microRNAs, cortical circular RNAs (circRNAs) are associated with AD diagnosis, clinical dementia severity, and neuropathological severity, suggesting the potentially significant role of circRNA in the pathogenesis of AD (137). CircRNA *KIAA1586* is significantly enriched in AD-associated biological processes and may be a novel risk factor in the pathogenesis of AD (138). CircRNA *circ_0000950* promotes neuron apoptosis, inhibits the production of neurite outgrowth, and increases the levels of inflammatory cytokine levels via sponging microRNA-103 in AD (139). *CircNF1-419* upregulates autophagy and reduces Aβ and tau expression by binding the proteins Dynamin-1 and adaptor protein 2 B1 (140). *Circ-AXL, circ-GPHN*, and *circ-PCCA* differ significantly between AD patients and normal controls by studying the circRNA expression profile in cerebrospinal fluid, which may be potential biomarkers in AD (141). In addition, 147 circRNAs are differentially expressed in different AD brain regions, most of which are found in the parahippocampal gyrus, supporting that circRNAs in the parahippocampal gyrus may be biomarkers in AD (142).

In the central nervous system, long ncRNAs (lncRNAs) are prevalent and play a critical role in the pathogenesis of AD (143). A conserved lncRNA called BACE1-antisense transcript (*BACE1-AS*) increases BACE1 mRNA stability and generates additional Aβ in AD patients as well as in APP transgenic mice, and knockdown of *BACE1-AS* improves memory (144, 145). The BACE1-AS level differs significantly between pre-AD and healthy controls as well as full-AD and healthy controls, indicating that BACE1-AS may be a potential biomarker of AD (146). LncRNAs, including *51A, 17A*, and *NDM29*, increase the formation of Aβ and contribute to the pathogenesis of AD (147). LncRNA *SOX21-AS1* is upregulated in the AD mouse model, and its inhibition reduces neuronal oxidative stress and suppresses neuronal apoptosis via the Wnt signaling pathway (148). LncRNA *BC200* levels are enhanced significantly in AD brains and involved in dendritic loss by regulating local protein synthesis (149). Brain-derived neurotrophic factor antisense RNA(*BDNF-AS*) is an lncRNA that represses BDNF expression. The inhibition of *BDNF-AS* results in neuronal growth and differentiation, which may be the novel pharmacological target in AD (150). LncRNA *Sox2OT* represses *Sox2* gene expression, involving neurogenesis and neuronal differentiation (151). In APP/PS1 mice, lncRNA *EBF3-AS* is upregulated in the hippocampus and related to neuron apoptosis by regulating EBF3 expression (152). LncRNA *NAT-Rad18* increases the likelihood of apoptosis under DNA damage-related stress via targeting *RAD18* (153). LncRNA *TUG1* promotes neuronal apoptosis in the hippocampus by increasing microRNA-15a levels and suppressing ROCK1 expression (154). In the SAMP8 mouse model, lifestyle, including diet, exercise, and environmental enrichment, results in epigenetic changes (20). In addition, 3112 differentially expressed lncRNAs are identified in the SAMP8 mouse model, most of which are intergenic and exon sense-overlapping (155). In the human AD brain, 16 age-associated and 13 gender-associated lncRNAs are identified; among them, lncRNAs *SNHG19, LINC00672, RNF144A-AS1, LY86-AS1*, and *LINC00639* are associated with the pathology of AD (156). Nuclear paraspeckle assembly transcript 1 (*NEAT1*), a lncRNA that is widely expressed in cells, is of great importance in various biological and pathological processes by mediating target genes' expression (157). *NEAT1* is involved in Aβ clearance by regulating the expression of endocytosis-related genes in AD (158). Additionally, in an APP/PS1 transgenic mouse model, *NEAT1* is increased and promotes the pathogenesis of AD via upregulating PTEN-induced putative kinase 1 (PINK1)'s ubiquitination and degradation, which provided a potential therapeutic strategy in AD (159) (Table 4).

**Table 4:** Non-coding RNAs in AD.

**Non-coding RNAs**	**Tissue type**	**Main findings**	**References**
*MicroRNA-29a/b-1*	AD patients postmortem sporadic brain	*MicroRNA-29a/b-1* could suppress BACE1 expression and was significantly decreased in AD	(112)
*MicroRNA-107*	Human temporal cortex samples	Decreased *microRNA-107* expression	(116)
*MicroRNA-298*	Primary human cell culture model	*MicroRNA-298* repressed the expression of BACE1, APP, Aβ40, and Aβ42	(120)
*MicroRNA-34a-5p* and *microRNA-125b-5p*	Serum samples of 27 AD patients	*MicroRNA-34a-5p* and *microRNA-125b-5p* reduced Aβ	(121)
*MicroRNA-125b*	Primary neurons	*MicroRNA-125b* caused tau hyperphosphorylation	(124)
*MicroRNA-132*	Primary mouse and human wild-type neurons	*MicroRNA-132* was associated with the lower levels of tau phosphorylation	(126)
*MicroRNA−219-5p*	Human postmortem brain tissues	*MicroRNA-219-5p* was increased and associated with tau phosphorylation in AD	(122)
*MicroRNA-125b*	Human postmortem brain specimens	*MicroRNA-125b* could promote the phosphorylation of tau and was enhanced in AD	(123)
*MicroRNA-346*	Primary human brain cultures	*MicroRNA-346* upregulated APP translation and Aβ production	(132)
*MicroRNA-644*	Human HEK293, HeLa cells, and mouse Neuro2A cells	*MicroRNA-644* downregulated the formation of Aβ	(133)
circRNA	Neuropathologically confirmed AD case and control brain tissues	CircRNA was associated with AD diagnosis, clinical dementia severity and neuropathological severity	(137)
*Circ-AXL, circ-GPHN* and *circ-PCCA*	Cerebrospinal fluid from AD patients and control subjects	*Circ-AXL, circ-GPHN* and *circ-PCCA* differed significantly between AD patients and normal controls	(141)
circRNA	Human postmortem brain samples	147 circRNAs were differentially expressed in different AD brain regions	(142)
BACE1-AS	Human postmortem brain samples	BACE1-AS could increase BACE1 mRNA stability and generate additional Aβ	(144)
51A, 17A, and NDM29	Postmortem AD brain samples and AD cerebrospinal fluid	17A, 51A, and NDM29 increase Aβ formation and/or the Aβ_42_/Aβ_40_ ratio	(147)
LncRNAs	Human postmortem brain samples	*SNHG19* and *LINC00672, RNF144A-AS1, LY86-AS1*, and *LINC00639* were associated with the pathology of AD	(156)
*BC200*	Human postmortem brain samples	*BC200* levels enhanced significantly in AD brains	(149)
*BDNF-AS*	Human and mouse cell lines	*BDNF-AS* repressed BDNF expression	(150)
*Sox2OT*	AD mouse cerebral cortex	*Sox2OT* repressed *Sox2* gene expression, involving in neurogenesis and neuronal differentiation	(151)
*EBF3-AS*	Hippocampus of APP/PS1 mice	*EBF3-AS* was upregulated in hippocampus and related to neuron apoptosis	(152)
*NAT-Rad18*	AD rat cortical neurons	*NAT-Rad18* increased the likelihood of apoptosis	(153)
*TUG1*	AD mice model	*TUG1* promoted neuronal apoptosis	(154)
LncRNAs	SAMP8 mice	3,112 differentially expressed lncRNAs were found in hippocampus	(155)
*NEAT1*	APPswe/PS1dE9 double transgenic mouse model	*NEAT1* is involved in Aβ clearance	(158)
*NEAT1*	APP/PS1 transgenic mice model	*NEAT1* promoted the pathogenesis of AD	(159)
*MicroRNA-124-3p*	N2a/APP695swe cells	*MicroRNA-124-3p* inhibited abnormal tau hyperphosphorylation	(127)
*MicroRNA-106b*	SH-SY5Y cells	*MicroRNA-106b* reduced tau phosphorylation	(128)
*MicroRNA-330*	C57 mice	*MicroRNA-330* reduced Aβ production and alleviated oxidative stress	(134)
*MicroRNA-138*	APP/PS1 mice	*MicroRNA-138* enhanced Aβ production and improved cognitive impairment	(135)
*MicroRNA-98*	AD mice model	*MicroRNA-98* improved oxidative stress and downregulated Aβ production	(136)
*MicroRNA-369*	3xTg-AD mice	*MicroRNA-369* was associated with tau hyperphosphorylation	(129)
*MicroRNA-425-5p*	AD and HEK293/tau cells	*MicroRNA-425-5p* overexpression promoted tau phosphorylation	(125)
*MicroRNA-326*	AD mice models	*MicroRNA-326* decreased tau hyperphosphorylation and improved cognitive functions	(130)
*MicroRNA-195*	ApoE4^+/+^ mice	*MicroRNA-195* diminished tau hyperphosphorylation and Aβ burden	(131)
*MicroRNA-31*	Hippocampus of 17-month-old AD triple-transgenic (3xTg-AD) female mice	*MicroRNA-31* was able to reduce the mRNA levels of BACE1	(115)
*MicroRNA-200a-3p*	Hippocampus of APP/PS1 and SAMP8 mice as well as in blood plasma from AD patients	*MicroRNA-200a-3p* could reduce the expression of BACE1 and confirmed to be decreased in AD	(113)
*MicroRNA-16*	Hippocampus of aged SAMP8 mice and murine cells	*MicroRNA-16* led to reduced APP protein expression and was decreased in AD mice	(111)
*MicroRNA-340*	Hippocampus of AD model SAMP8 mouse	*MicroRNA-340* was decreased and associated with the overproduction of Aβ	(114)
*BACE1-AS*	SAMP8 mice	Knockdown of BACE1-AS inhibited BACE1 and improved memory	(145)
*SOX21-AS1*	AD mice model	LncRNA SOX21-AS1 was unregulated and resulted inneuronal oxidative stress in AD	(148)
*MicroRNA-195*	SAMP8 mice and HEK293 cells	SAMP8 mice and HEK293 cells	(119)
*MicroRNA-124*	Cellular AD model	*MicroRNA-124* was steadily altered and associated with BACE1	(117)
*MicroRNA-298* and *microRNA-328*	Neuronal (N2a) and fibroblastic (NIH 3T3) cells	*MicroRNA-298* and *microRNA-328* were associated with BACE1	(118)
circRNA *circ_0000950*	Cellular AD model	C*irc_0000950* promoted neuron apoptosis, inhibited the production of neurite outgrowth, and increased the levels of inflammatory cytokines levels	(139)
circRNA *CircNF1-419*	SD rat model	*CircNF1-419* upregulated autophagy and reduced Aβ and tau	(140)

*circRNA, circular RNA; BACE1-AS, BACE1-antisense transcript; SOX21-AS1, SOX21 antisense RNA 1; NEAT1, Nuclear paraspeckle assembly transcript 1*.

The mechanisms underlying epigenetic regulation of non-coding RNAs in AD are complex. As we mention, circRNAs mediate the effect of microRNAs (139). MicroRNAs are regulated by chromatin modifications and DNA methylation, which are implicated in AD by targeting messenger RNA (mRNA) expression (182). Dysregulated circRNAs are associated with the increased number of downstream target mRNAs in the Tg2576 AD mouse model, indicating that circRNA-microRNA-mRNA may play a significant role in the pathogenesis of AD (183). Emerging evidence shows that lncRNAs are involved in multiple epigenetic processes, such as DNA methylation, via regulating the interactions of target genes with chromatin-remodeling enzymes. Most lncRNAs are located in the nucleus, in which they work as scaffolds for chromatin modifiers or transcriptional co-regulators to exhibit regulatory functions (184). In addition, lncRNAs can alter transcription, mRNA stability, alternative splicing, and translational activity in AD, resulting in aberrant gene expression (14). Overall, the deregulated and complex non-coding RNAs are closely associated with core pathophysiological processes of AD via regulating gene expression at different levels, including transcription, RNA processing, and translation (185).

## Epigenetic Therapeutic Strategies for AD

DNA hypomethylation of pathogenetic genes is associated with the overproduction of Aβ (186). Oliveira et al. find that DNA methyltransferase Dnmt3a2 is decreased in the hippocampus of mice, and the restoration of Dnmt3a2 recovered the cognitive functions (160, 187). Further, betaine, a methyl donor, ameliorates the memory deficits in mice (161). Another methyl donor, S-adenosylmethionine (SAM), is decreased in the cerebrospinal fluid of AD patients (188). SAM reduces the production of Aβ and tau phosphorylation by upregulating PSEN1 and BACE1 expression *in vivo*, which improves the cognitive status in AD mouse models (162). The treatment with alcohol extracts from G. lucidum increases methylation regulators and improves memory in APP/PS1 AD model mice (163). In AD and mild cognitive impairment patients, B vitamin intake results in hypermethylation of *NUDT15* and *TXNRD1*, which is associated with better cognitive performance (164). The supplementation of folic acid, a methyl donor, improves cognitive functions in participants who tend to decline with age (165). Interestingly, maternal supplementation of resveratrol promotes cognitive decline in the SAMP8 mice offspring via increasing global methylation levels and decreasing hydroxymethylation levels (166). Consequently, the DNA methyltransferase or the methyl donor may be potential treatments for patients with AD.

Meanwhile, hypermethylation is involved in the development of AD as well; thus, the decrease of methylation levels in some genes may also be a promising therapeutic strategy (109). DNMT inhibitors are used in the treatment of hematopoietic malignancy (189). Also, the administration of DNMT inhibitors is used in some neurodegenerative diseases, such as Friedreich's ataxia (190). Although DNMT inhibitors possess the potential for the therapy of AD, the lack of gene-specificity and security is the main difficulty needed to resolve before its use in AD patients (177). As we previously mentioned, the epigenetic markers are altered in the 5xFAD mouse model. Treatment of UNC0642 inhibits the methyltransferase activity G9a/GLP and restores cognition by reducing 5mC and increasing 5hmC in the 5xFAD mouse model (167). The knockout of the *Tet1* gene enhances cognitive function by oxidizing 5 mC to 5 hmC and reducing methylation levels of the brain in mice (191).

Histone deacetylases (HDACs) inhibit gene expression and are associated with memory impairment by restricting access of transcription factors to memory storage-related genes (192). HDAC inhibitors (HDACi) are considered to be the potential therapeutic strategy in AD. Trichostatin A, an HDACi, restores contextual freezing performance and H4 acetylation levels in the APP/PS1 mouse model of AD (100). Valproic acid (VPA) is one of the first discovered HDACi and beneficial in memory enhancement in the mouse model of AD (168). Histone deacetylase 2 (HDAC2) inhibitors improve memory by promoting the formation and growth of dendritic spines in mice (193, 194). Sodium phenylbutyrate, an HDACi, also alleviates memory impairment in transgenic AD mice by inducing neurotrophin expression via the protein kinase C (PKC)-cAMP-response element-binding protein (CREB) pathway (169). M344, an HDACi, lowers the expression of Aβ and prevents cognitive decline with the normalization of several pathogenic pathways in triple transgenic (APPsw/PS1M146V/TauP301L) mice *in vivo* (170). A mercaptoacetamide-based class II HDACi and a hydroxamide-based class I and II HDACi reduce Aβ levels *in vitro* and rescue memory loss in AD mice (171). The downregulated PU.1 expression is associated with lower AD risk in a genome-wide association study (195). High-throughput screening of FDA-approved drugs reveals a HDACi, vorinostat, decreases PU.1 expression and may be a useful therapeutic approach in AD (172). Moreover, the administration of RGFP-966, a selective HDAC3 inhibitor, improves cognitive function in the AD mouse model and decreases Aβ and tau in neurons from AD patients, further supporting the significant role of HDACi in patients beyond the AD mouse model (173). Therefore, HDACi can be seen as a potential therapeutic agent for AD. However, HDACi is widely targeted and inevitably causes various side effects, including apoptosis and arrest of the cell cycle; therefore, successful experiments in AD animal models are seldom feasible for clinical application in humans. To increase the sensitivity of HDACi is a critical issue in the future (196). Moreover, histone acetyltransferase (HAT) is involved in the formation of CREB binding protein (CBP), which has an important role in memory (197). The expression of CBP could help transgenic AD mice recover memory impairment. The activator of histone acetyltransferases CBP/p300 is capable of passing the blood–brain barrier and extending the recent memory duration in B57BL6/6J male mice *in vivo*, which may be a potential treatment target in AD (174). In the late-stage FAD mouse model, the inhibitors of euchromatic histone methyltransferases decrease histone hypermethylation and improve synaptic deficits and cognitive functions, providing a possible novel therapeutic strategy for AD (175).

Noncoding RNAs are also involved in the pathogenesis of AD. The decreased expression of BACE1 is obtained by short-interfering RNA, resulting in the reduction of Aβ and tau phosphorylation levels in AD transgenic mice (176). The expression of APP, PSEN1, and PSEN2 was downregulated by RNA interference, such as short-interfering RNA and short hairpin RNA, which provide a promising therapeutic strategy in the future (177). MicroRNA mimics and anti-microRNAs are being developed by decreasing target protein expression in AD. *MicroRNA-384* mimic downregulating the expression of APP and BACE1 in SH-SY5Y cells, demonstrating that *microRNA-384* may be a potential target in AD (178). Anti-microRNAs complement respective microRNAs and reduce their levels to restore homeostasis. In the AD mouse model, *microRNA-146a* is upregulated, and anti-microRNA-146a-base treatment improves cognitive functions and regulates the inflammatory response by using the viral vector delivery system (179). In addition, a number of other microRNAs are the potential treatment targets in AD. *MicroRNA-34c* increases in the hippocampus and blood of patients with AD, and its inhibitor enhances memory in AD mice models *in vivo* (180). LncRNA *BACE1-AS* is positively associated with BACE1 protein expression *in vitro* and *in vivo*, and knockdown of *BACE1-AS* by short interfering RNA improves cognitive function in a mouse model of AD (145). *MicroRNA-339-5p, microRNA-29c*, and *microRNA-124* decrease BACE1 expression *in vitro* (181, 198, 199) (Table 5). *MicroRNA-101* suppresses APP and Aβ expression in hippocampal neurons *in vitro* (200). *MicroRNA-153* decreases APP expression in primary human fetal brain cultures (201). Consequently, non-coding RNAs may be potential targets for AD therapy in the future. Currently, there are several problems in the treatment of AD through non-coding RNAs, including altering such targets, off-target effects, and delivery methods (202). However, it is worthwhile to investigate non-coding RNAs in AD by appropriate understanding and safe manipulation (203).

**Table 5:** Epigenetic therapeutic strategies in AD.

**Therapeutic strategies**	**Model**	**Main findings**	**References**
Dnmt3a2 (DNA methyltransferase)	Aged mice	The raise Dnmt3a2 level in the hippocampus of aged mice enhanced cognitive ability	(160)
Betaine(a methyl donor)	Male ddY strain mice	Betaine treatment ameliorate memory deficit	(161)
S-adenosylmethionine(SAM, a methyl donor)	TgCRND8 mice	SAM reduced the Aβ production and improved the memory	(162)
Alcohol extracts from G. lucidum	APP/PS1 AD model mice	It increased methylation regulators and improved memory	(163)
B vitamin	AD patients and mild cognitive impairment patients	It resulted in hypermethylation of *NUDT15* and *TXNRD1* and better cognitive performance	(164)
Folic acid	Participants who tend to decline with age	The supplementation of folic acid improved cognitive functions	(165)
Resveratrol	SAMP8 mice	It promoted cognitive decline in the SAMP8 mice offspring	(166)
UNC0642	5XFAD mouse model	It inhibited the methyltransferase activity G9a/GLP and restored cognition	(167)
Trichostatin A	AD mouse model	It restored contextual freezing performance	(100)
Valproic acid (VPA, a histone deacetylase inhibitor)	APPswe/PS1ΔE9 (APP/PS1) transgenic mice	VPA decreased Aβ deposition and increased memory ability	(168)
Sodium phenylbutyrate (a histone deacetylase inhibitor)	5XFAD mice	Sodium phenylbutyrate improved memory and spatial learning	(169)
M344 (a histone deacetylase inhibitor)	Triple transgenic (APPsw/PS1M146V/TauP301L) mice	M344 lowered the expression of Aβ and prevent cognitive decline	(170)
Mercaptoacetamide-based class II HDACi and a hydroxamide-based class I and II HDACi	3xTg AD mice	They reduced Aβ levels *in vitro* and rescued memory loss	(171)
Vorinostat	Primary human brain tissue	It decreased PU.1 expression and was associated with lower AD risk	(172)
RGFP-966	AD mice model	It improved cognitive functions	(173)
CBP/p300 (an activator of histone acetyltransferase)	3xTg-AD mice	CBP/p300 extended the recent memory duration	(174)
The inhibitors of euchromatic histone methyltransferases	FAD mouse model	It decreased histone hyper-methylation and cognitive functions	(175)
Short-interfering RNA	AD mice models	It decreased the expression of BACE1	(176)
RNA interference	AD mice models	It downregulated the expression of APP, PSEN1, PSEN2	(177)
*MicroRNA-384* mimic	SH-SY5Y cells	*MicroRNA-384* mimic downregulated the expression of APP and BACE1	(178)
Anti-microRNA-146a-base treatment	AD mouse model	It improved cognitive functions in AD	(179)
Inhibitor of *microRNA-34c*	AD mice models	It could enhance the memory ability	(180)
Knockdown of BACE1-AS by lentivirus	SAMP8 mice	It improved learning behaviors and memory	(145)
*MicroRNA-124*	SH-SY5Y cells	It decreased apoptosis and decreased Aβ-induced viability inhibition	(181)

*HDACi, inhibitor of histone deacetylase; BACE1-AS, BACE1-antisense transcript; NUDT15, nudix hydrolase 15; TXNRD1, thioredoxin reductase 1*.

Currently, a few drugs are available for the treatment of AD. No drug can cure or stop disease development. Given the huge number and seriousness of AD, the need for clinical trials is necessary. To date, most of the clinical trials of AD have targeted Aβ, tau protein (204). Several clinical trials investigate epigenetics for the treatment of AD. Oral betaine was given in eight AD patients; however, the efficacy of betaine could not be determined due to the lack of controls and small sample size (205). With the use of S-adenosylmethionine and nutriceutical, almost 30% improvement in the neuropsychiatric inventory and activities of daily living is observed in AD patients compared to normal controls (206). RDN-929, a selective HDAC inhibitor, is being investigated in a phase I clinical trial for the treatment of AD patients. The result is not disclosed at clinicaltrials.gov. EVP-0334, also called FRM-0334, a CNS-penetrant HDACi, phase I testing of AD is completed; however, no result is posted (207). Because increasing evidence shows that epigenetics plays an essential role in AD, the drugs related to epigenetics may be breakthroughs for AD in the future.

## Conclusion

To date, although some genetic and non-genetic factors are well-studied, the pathogenesis of AD remains unclear. Epigenetics provides us with an important insight into how AD develops. There is an increasing number of studies about epigenetics in AD patients, including DNA methylation/hydroxymethylation, histone modifications, and non-coding RNAs. Epigenetic genome-wide association (EGWA) studies show that many differentially methylated sites exist in AD compared with normal controls. Several studies investigate the role of histone modifications in AD. Non-coding RNAs play an important role in the pathogenesis of AD. LncRNAs, such as *BACE1-AS*, increases BACE1 mRNA stability and generates additional Aβ in AD. These studies show us that epigenetics is of great importance in AD, suggesting that epigenetics can be a potential intervention target in treating AD given the reversible nature of epigenetic changes. Therapeutic attempts include the use of inhibitors of HDACs, DNA methyltransferase, and inhibitors of non-coding RNAs, which have shown some exciting results in animal studies. Despite the numerous and exciting findings of epigenetics in AD, the results are less satisfying. The data is often controversial and lacks definite results. There is a need to design some larger longitudinal cohorts to study the epigenetic changes of AD, which may help us better understand the pathogenesis of AD and find novel strategies to treat AD in the future.

## Author Contributions

BJ was involved in the review design, modified, and revised the manuscript. XX and XL searched and reviewed the articles. XX wrote the manuscript. All authors contributed to the article and approved the submitted version.

## Conflict of Interest

The authors declare that the research was conducted in the absence of any commercial or financial relationships that could be construed as a potential conflict of interest.
